# Application of Fourier transform infrared spectroscopy with chemometrics on postmortem interval estimation based on pericardial fluids

**DOI:** 10.1038/s41598-017-18228-7

**Published:** 2017-12-21

**Authors:** Ji Zhang, Bing Li, Qi Wang, Xin Wei, Weibo Feng, Yijiu Chen, Ping Huang, Zhenyuan Wang

**Affiliations:** 1 Shanghai Key Laboratory of Forensic Medicine, Shanghai Forensic Service Platform, Academy of Forensic Science, Ministry of Justice, Shanghai, 200063 China; 2 Department of Forensic Pathology, Xian Jiaotong University, Xi’an, Shaanxi 710061 China; 3 Qingpu Branch of Shanghai Municipal Bureau of Public Security, Shanghai, 201799 China; 4 Cadet Brigade, The Fourth Military Medical University, Xi’an, Shaanxi 710032 China

## Abstract

Postmortem interval (PMI) evaluation remains a challenge in the forensic community due to the lack of efficient methods. Studies have focused on chemical analysis of biofluids for PMI estimation; however, no reports using spectroscopic methods in pericardial fluid (PF) are available. In this study, Fourier transform infrared (FTIR) spectroscopy with attenuated total reflectance (ATR) accessory was applied to collect comprehensive biochemical information from rabbit PF at different PMIs. The PMI-dependent spectral signature was determined by two-dimensional (2D) correlation analysis. The partial least square (PLS) and *nu*-support vector machine (*nu*-SVM) models were then established based on the acquired spectral dataset. Spectral variables associated with amide I, amide II, COO^−^, C-H bending, and C-O or C-OH vibrations arising from proteins, polypeptides, amino acids and carbohydrates, respectively, were susceptible to PMI in 2D correlation analysis. Moreover, the *nu*-SVM model appeared to achieve a more satisfactory prediction than the PLS model in calibration; the reliability of both models was determined in an external validation set. The study shows the possibility of application of ATR-FTIR methods in postmortem interval estimation using PF samples.

## Introduction

Postmortem interval (PMI) evaluation remains a challenge in the forensic community because routine methods rely on subjective evaluation of body signs alone during the early phase (usually within 24 h postmortem), including algor mortis, livor mortis, rigor mortis distribution, and corneal turbidity^1^. In recent years, an increasing number of investigations have focused on postmortem chemical changes in biofluids, especially the vitreous humor and blood^2,3^, to identify biomarkers for PMI estimation, since they are readily available at crime scenes or during autopsy. There is ample evidence that multiple components in pericardial fluid (PF), including heart-specific proteins (cardiac troponin and creatine kinase MB), mRNAs, and electrolytes (Ca^2+^ and Mg^2+^), may be used to determine specific causes of death and elucidate the underlying mechanisms^4–6^. However, the potential of PF as a medium for PMI determination has not been documented sufficiently. Only a few studies indicate that electrolytes in PF tend to be used as parameters for PMI estimation. For instances, Balasooriya et.al showed that changes in K^+^, phosphates, and Na^+^ concentrations are significantly correlated with PMI^7^. Subsequently, Dalbir *et al*. established mathematical models based on electrolytic parameters for PMI prediction in independent samples^8^. Nevertheless, limited variables in PF are taken into account in the above studies; in addition, there is no evidence that other substances could contribute to sequential postmortem changes.

Fourier transform infrared (FTIR) spectroscopy is a powerful analytical tool for identifying chemical constituents and elucidating compound structures in various forms in real-world samples according to the vibrational modes of their molecular functional groups^9,10^. FTIR has the capacity to perform global assessment of components found in samples with no need of sample preparation, which is practically impossible with other routine analytical approaches. In forensic investigations, FTIR has been extensively utilized in multiple evidence-based cases at a crime scene, including questioned documents^11^, banknotes^12^, paints^13^, fibers^14^, hair^15^ and gunshot residues^16^. Alternatively, the feasibility of FTIR for chemically analyzing biological specimens has been demonstrated by other studies; indeed, multiple macromolecules, such as proteins, lipids, carbohydrates, and nucleic acids, can be monitored simultaneously in an FTIR spectrum based on their unique infrared absorption frequencies^17,18^. However, due to the complexity and heterogeneity of biological systems, a variety of data processing methods have emerged to interpret and select spectral features. In this context, two-dimensional (2D) correlation analysis is commonly used to uncover overlapped bands and discriminate very complex mixtures under the conditions of external perturbations, such as time, temperature, concentration and oxidation^19–23^. Moreover, a combination of FTIR spectroscopy and chemometric methods, including partial least square (PLS) and support vector machine (SVM) models, can convert the characteristic spectral pattern into a classifier or discriminator for automatic classification and prediction among different sample categories^24,25^.

In our research team, much efforts have been devoted to characterizing postmortem changes in biological samples by FTIR spectroscopy. We found that some spectral parameters, e.g. peak intensities and areas, are correlated with PMI in different tissues^26,27^. Recently, PMI groups of the rabbit plasma are successfully distinguished by FTIR spectroscopy coupled with PLS models^28^. The present study primarily focused on PF due to its advantages. For instance, large amounts of PF are easily obtained in contrast to VH; meanwhile, PF is less susceptible to microbial contamination and bacterial degradation compared with blood samples. To the best of our knowledge, this is the first study of PMI estimation based on infrared spectroscopic analysis of PF.

## Materials and Methods

### Animal model

A total of 99 male Japanese rabbits (6 months; 2.5–2.8 kg) were purchased from the animal center of Xian Jiaotong University. They were socially housed under a 12 h light/dark cycle with food and water *ad libitum*. The animal experiments were approved by the Committee of Laboratory Care and Use of Xian Jiaotong University. All methods were performed in accordance with the relevant guidelines and regulations outlined by the Committee of Laboratory Care and Use of Xian Jiaotong University. The rabbits were sacrificed by air injection through the ear-rim vein, and carcasses were placed in isolated chambers at a constant temperature of 25 °C. PF samples were harvested from the pericardium using sterile syringe needles within 48 h postmortem at 6 h intervals (11 rabbits per time point). The samples were then immediately submitted to centrifugation at 14000 rpm for 10 minutes to eliminate particle matters, which may cause Mie-type scattering. The supernatants were obtained and snap frozen in liquid nitrogen until use for FTIR analysis. The animals were randomly divided into calibration (8 rabbits per group) and validation (3 rabbits per group) groups.

### FTIR measurements

Spectroscopic measurements were performed on a Nicolet IS 50 FTIR spectrometer(Thermo Scientific Fisher, USA) coupled with an ATR accessory (Smart Orbit Diamond, Thermo Scientific Fisher, USA). When an infrared beam is directed onto the ATR diamond crystal with a high refractive index, the generated evanescent waves penetrate a few microns on the sample surface and are subsequently attenuated or altered due to energy absorption. ATR-FTIR measures such energy variation for selected wavelengths, and produces corresponding infrared spectra. Peak intensity and position in an infrared spectrum are primarily dependent upon global vibrational modes of molecular functional groups in a given sample. In this study, the laboratory environment was kept at a temperature of 37 °C, with a relative humidity below 20%, in order to remove atmospheric water vapor as much as possible. Before FTIR measurements, approximately 100 μL of the thawed sample was shaken on a vortex mixer for 30 s and mixed with a micropipettor. Next, a sample aliquot (1 μL) was carefully deposited on the ATR diamond window and sufficiently dried with an air dryer. Spectra were collected at frequencies ranging from 1800 to 900 cm^−1^, with a resolution of 4 cm^−1^ and 32 scans. Background spectra collected on blank ATR spectra were automatically subtracted. For each sample, nine replicates were automatically averaged to produce a spectrum in order to eliminate loading errors.

### Two-dimensional (2D) correlation analysis

All FTIR spectra in each PMI group were averaged. Average spectra in all groups were normalized by SNV and analyzed by the 2Dshige software package (Shigeaki Morita, Osaka Electro-Communication University, Japan; version 1.3).

### Chemometrics

PLS and *nu*-SVM regression models were established with MATLAB R2014a (MathWorks, USA). Spectral datasets were preprocessed by SNV and second derivatives (25 points smoothing) within a frequency window of 1800–900 cm^−1^. The predictor X corresponded to the matrix of spectral intensity while the response variable Y was associated with PMI values. To reduce computational complexity in establishing a *nu*-SVM model, the dimensions of preprocessed spectra were reduced to 8 latent factors by principal component analysis (PCA). This method can transform a high dimensional dataset into a lower dimensional orthogonal feature set while retaining maximum information from the original high dimension dataset^29,30^. In this study, these 8 latent factors explained rough 98% of the variance. The calibration dataset was used to establish mathematical models. Their reliability was evaluated by 8-fold cross-validation and a permutation test to avoid overfitting, which usually renders models impractical in predicting independent samples accurately. In 8-fold cross-validation, the calibration dataset was divided into 8 equal sized sub-datasets, each of which contained spectra from 9 PMI groups. Of the 8 sub-datasets, one was retained as the test dataset, and spectral categories in this sub-dataset were predicted by the model established using the remaining sub-datasets. This process was repeated 8 times, and the determination coefficient (R^2^) and root-mean-square error of cross-validation (RMSECV) were assessed each time; these parameters represented the goodness of fitting between actual and predictive PMI values, and the global predictive error, respectively. Performances of the PLS and *nu*-SVM models were compared by unpaired *t*-test based on R^2^ and RMSECV, using Prizm 5.0 (GraphPad Software Inc., La Jolla, CA). *P* < 0.05 was considered statistically significant. Data were expressed as mean ± standard deviation (SD). In the next step, the established PLS and *nu*-SVM models were used to estimate PMI values in the validation group. Determination coefficient (Q^2^) and root-mean-square of prediction (RMSEP) values were also calculated to evaluate the generalization of the above models.

## Results and Discussion

**Figure 1:**
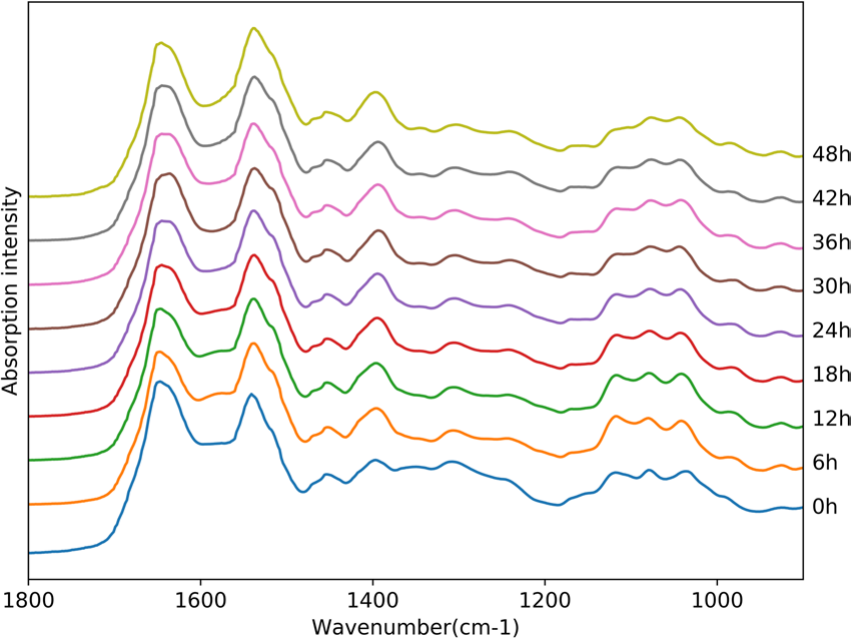
A comparison of average spectra with SNV normalization among PMI groups from 0 to 48 h postmortem.

Figure 1 shows a comparison of average spectra with SNV normalization from 1800–900 cm^−1^ among different PMI groups; the absorption bands were mainly associated with proteins, lipids, nucleic acids, and carbohydrates. According to previous studies^29–34^, molecule assignments are summarized in Table 1. The two most prominent peaks were related to proteins, including a band at around 1650 cm^−1^ arising from amide I (mostly the C=O stretching vibrations of the peptide back bone) and a band at around 1540 cm^−1^ assigned to amide II (N-H bending coupled with C-N stretching). Both bands are highly sensitive to conformational changes, and several absorption peaks related to protein secondary structures contribute to the peak shapes of amide I and II bands. The band at around 1453 cm^−1^ originated from asymmetric and symmetric C-H bending modes of proteins, while that at 1398 cm^−1^ resulted from COO^−^ vibrations of fatty acids, amino acids and polypeptides. In the frequency range of 1200–900 cm^−1^, the band at 1078 cm^−1^ was assigned to symmetric phosphate (*vs*PO_2_^−^) and C-O stretching modes of nucleic acids and carbohydrates, respectively, while those at around 1033 and 926 cm^−1^ resulted from C-O or C-OH vibrations of carbohydrates. In this study, vibrations by fatty and nucleic acids are negligible because the PF contains only low levels of both macromolecule types; thus, their functional groups are not detected by ATR-FTIR spectroscopy. Although multiple substances can be identified globally in spectral profiles, PMI groups cannot be distinguished based only on these spectra.

**Table 1:** FTIR frequencies of measured range and their peak assignment.

**Position [cm** ^**−1**^ **]**	**Assignment**
1650	Amide I: C=O stretching of the peptide back bone
1540	Amide II: N-H bending coupled to C-N stretching
1453	Asymmetric and symmetric C-H bending from CH_2_ and CH_3_ on proteins
1398	C=O vibrations of COO^−^ from free fatty acids, free amino acids and polypeptides
1324	Amide III from proteins
1078	Symmetric stretching of P-O from nucleic acids and phospholipids; C-H or C-OH vibrations from saccharides.
1033	C-O or C-OH vibrations from glucose, polysaccharides
926	C-O or C-OH vibrations from carbohydrates

**Figure 2:**
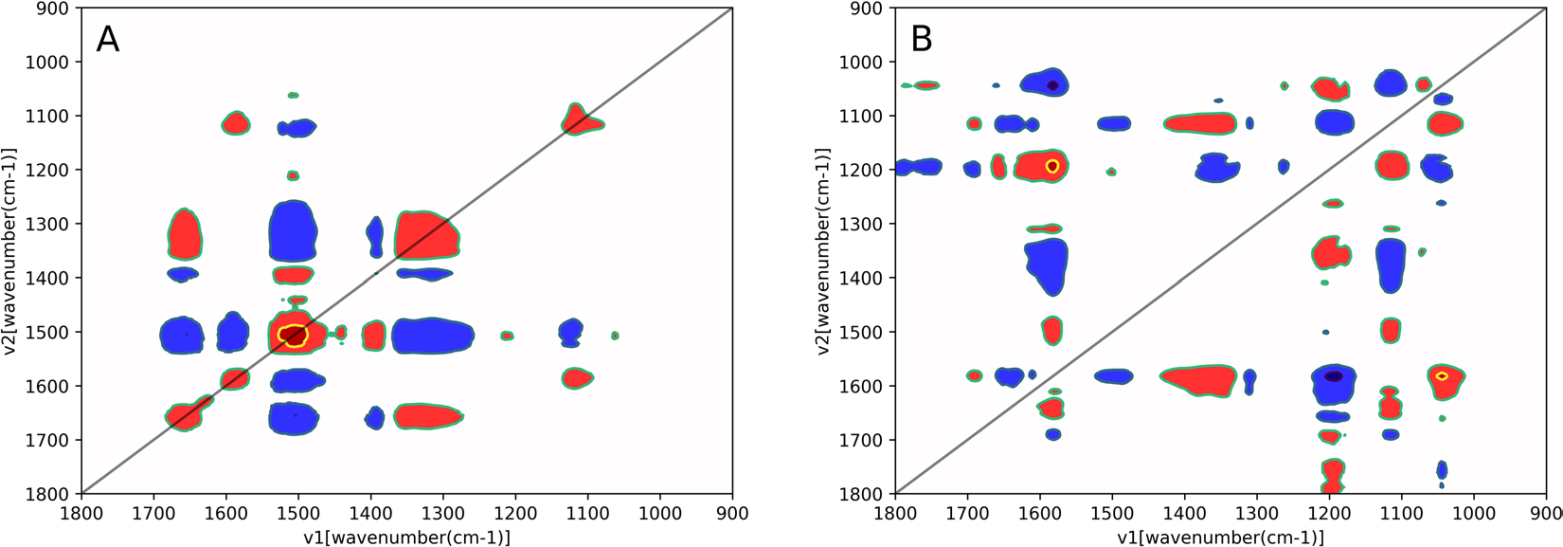
The results of 2D correlation analysis include synchronous (**A**) and asynchronous spectral maps (**B**).

Minor differences in spectra among PMI groups were elucidated by 2D correlation analysis. The latter method provides a robust analysis of kinetic changes in spectral data resulting from external perturbation such as temperature, concentration and oxidation, and determines whether spectral changes are correlated as well as the order of chemical changes in samples. In this study, the external perturbation was biochemical changes in the PF with PMI development. The results of 2D correlation analysis showed two types of correlation spectra, including synchronous (Φ (ν_1_, ν_2_)) and asynchronous (Ψ (ν_1_, ν_2_)) spectra (Fig. 2). In synchronous spectra (Fig. 2A), the auto-peaks at the diagonal line corresponded to the autocorrelation function of spectral intensity variations due to postmortem disturbance. The stronger the intensities of such peaks, the more sensitive they are to postmortem changes. The cross-peaks at the non-diagonal line provided information on relative correlations between pairs of spectral variables; positive features (red) were in the same direction, and negative (blue) ones in the opposite direction. In contrast, asynchronous spectra (Fig. 2B) showed the sequence of kinetic changes, with cross-peaks corresponding to counterparts in the synchronous spectral map. According to Noda’s rules^35^, when cross-peak signals are the same for both synchronous and asynchronous maps, intensity changes of spectral variables on the x-axis occur before those on the y-axis, and vice versa.

The results of 2D correlation analysis are summarized in Table 2. The most sensitive variables were mainly associated with proteins as strongest auto-peaks were found at 1656, 1581, and 1517 cm^−1^, all of which represent various protein secondary structures in the amide I and II structures. A broad auto-peak was found at around 1324 cm^−1^, involving amide III of proteins as well as COO^−^ vibration from amino acids and polypeptides, while a band at around 1089 cm^−1^ corresponded to C-O or C-OH vibrations from carbohydrates. These findings demonstrated that spectral changes at 1656, 1517, and 1324 cm^−1^ were found simultaneously before those at 1581 and 1089 cm^−1^, but chemical changes in both stages showed no significant correlation with PMI progression. In the early stage, the band at 1656 cm^−1^ is considered one of the characteristic absorption peaks of blood cells^36^. We assume that postmortem infiltration of hemoglobin and/or other proteins with similar structures into the PF may be responsible for such signal, resulting in color change of the PF from clear to dark red. The bands at around 1517 and 1324 cm^−1^, both of which had an opposite variation direction, reflected the process of protein degradation into various amino acids and polypeptides. The spectral variations at around 1581 (from proteins) and 1089 cm^−1^ (from carbohydrates) likely derived from specific glycoproteins leaked into the PF as the biological barriers collapse thoroughly at the later stage.

**Table 2:** Signs of the synchronous (Φ) and asynchronous (Ψ) cross peaks^a^.

(ν_1_, ν_2_)	Φ	Ψ	‘Sequential order’
(1089, 1656)	n	+	no correlation
(1324, 1656)	+	n	1324 = 1656
(1517, 1656)	−	n	1517 = 1656
(1581, 1656)	n	+	no correlation
(1089, 1581)	+	n	1089 = 1581
(1324, 1581)	n	+	no correlation
(1517, 1581)	−	−	1517 > 1581
(1089, 1517)	−	+	1089 < 1517
(1324, 1517)	−	n	1324 = 1517
(1089, 1324)	n	−	no correlation

^a^‘n’ means no cross peaks in the synchronous and asynchronous maps. Greater-than and less-than signs represent that ν_1_ occurs before (>) or after (<) ν_2_ respectively. The equal sign means that ν_1_ coincides with ν_2_.

**Figure 3:**
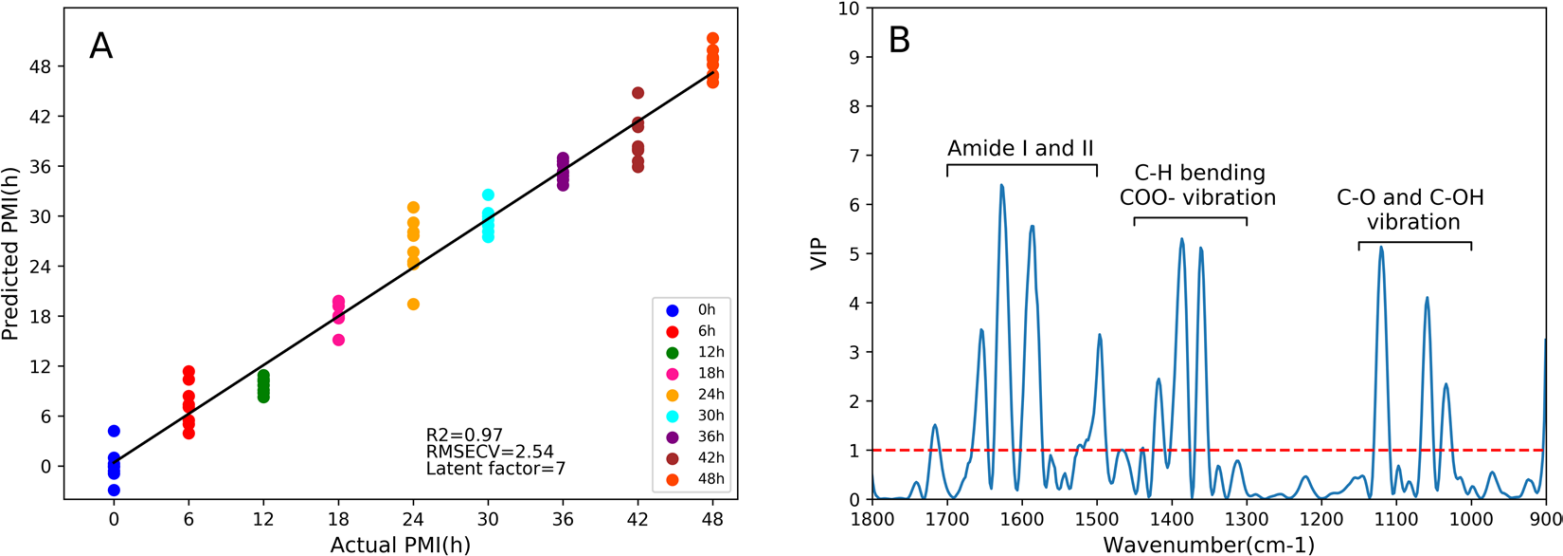
The cross-validation results of the PLS model using spectral variables within 1800–900 cm^−1^. (**A**) The regression plot between the predicted and actual PMI. The black line represents the reference line where the predicted PMI scores are closer to it, the higher fitting of goodness will be. (**B**) The plot of VIP scores displays the contribution of the spectral variables to the distinction in the PLS model. The variables with VIP scores above 1.0 (marked by a red dot line) are considered most significant, and their assignments are symbolized.

In the next step, chemometrics was employed to estimate PMI according to the FTIR spectral dataset. The PLS algorithm can extract principal components (referred to as latent factors) simultaneously from the predictor X and the response variable Y to construct a predictive model. Figure 3A demonstrates that the PLS model using 7 latent factors yielded a relatively satisfactory result (R^2^ = 0.97 ± 0.0067; RMSECV = 2.54 ± 0.45) in 8-fold cross-validation. Furthermore, this model was interpreted by calculating the variable importance in projection (VIP) for all spectral variables, the weighted sum of squares of the PLS weights^37^. Predictors with VIP values above 1.0 were considered influential variables for distinguishing PMI groups. The larger the VIP value for each variable, the more important the variable to the PLS model. In Fig. 3B, the main influential variables arose from proteins and related degradation products (including Amide I, Amide II, C-H bending and COO^−^ vibrations), followed by C-O or C-OH vibrations from carbohydrates. This finding further highlights the importance of protein degradation in the PF for PMI estimation.

**Figure 4:**
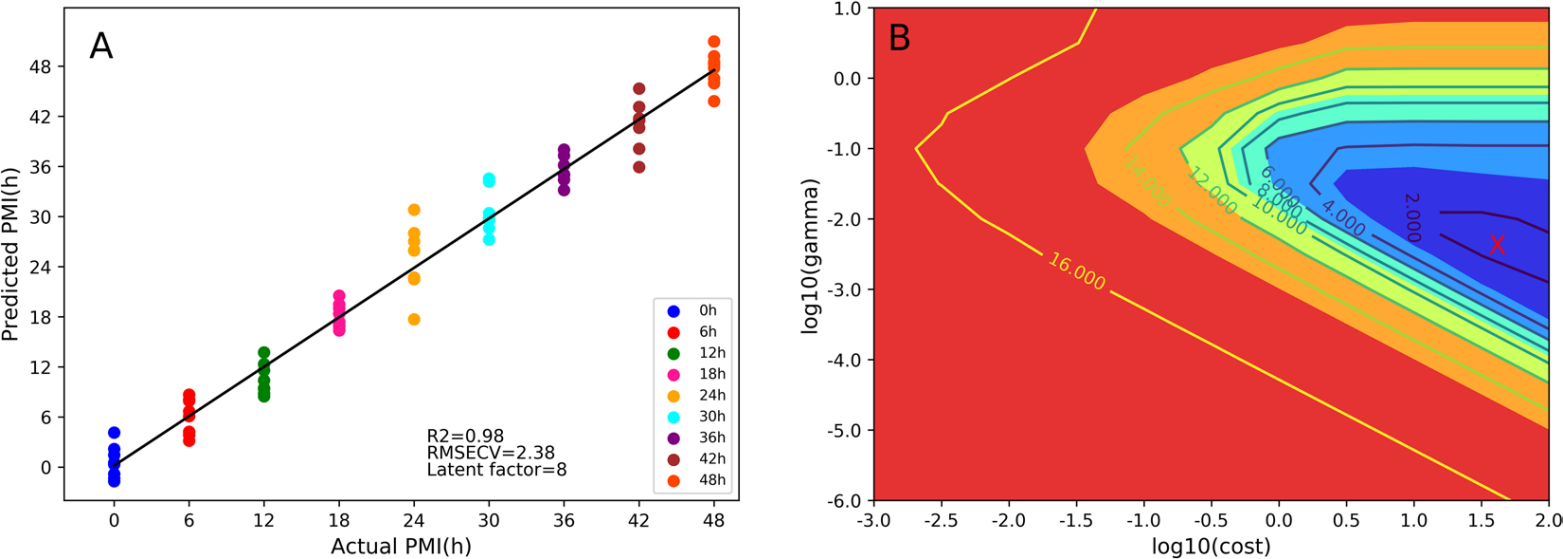
The cross-validation results of the *nu*-SVM model using spectral variables within 1800–900 cm^−1^. (**A**) The regression plot between the predicted and actual PMI. The black line represents the reference line. (**B**) The optimal combination of *nu*-SVM parameters, including *gramma* and *cost* is marked by the position of the red sign “X” where the minimum error can be obtained.

In Fig. 4A, the *nu*- SVM model achieved a better prediction with higher R^2^ value (0.98 ± 0.0082) and lower RMSECV (2.38 ± 0.42) compared with the PLS model (R^2^, *P* < *0.05*; RMSECV, *P* < *0.05*). This may be due to the ability of the *nu*-SVM model to avoid difficulties of using linear functions in high dimensional feature space. Indeed, *nu*-SVM regression introduces the new penalty parameter *nu*, which was set to 0.02 in this study. This parameter controls the number of support vectors and training errors. In SVM regression, only support vectors were used for the final PMI estimation. A Radial Basis Function (RBF) kernel with parameter *cost* and *gamma* was selected for non-linear transformation that maps observations into a high-dimensional space. The parameter *gramma* is a regulation constant that affects the generalization performance of the *nu*-SVM model, while *cost* refers to the cost factor, which controls the balance between calibration errors and model complexity. The best combination of the parameters *gramma* and *cost* was determined according to the minimal predictive error of cross-validation in the form of a two-dimensional grid search (Fig. 4B).

**Figure 5:**
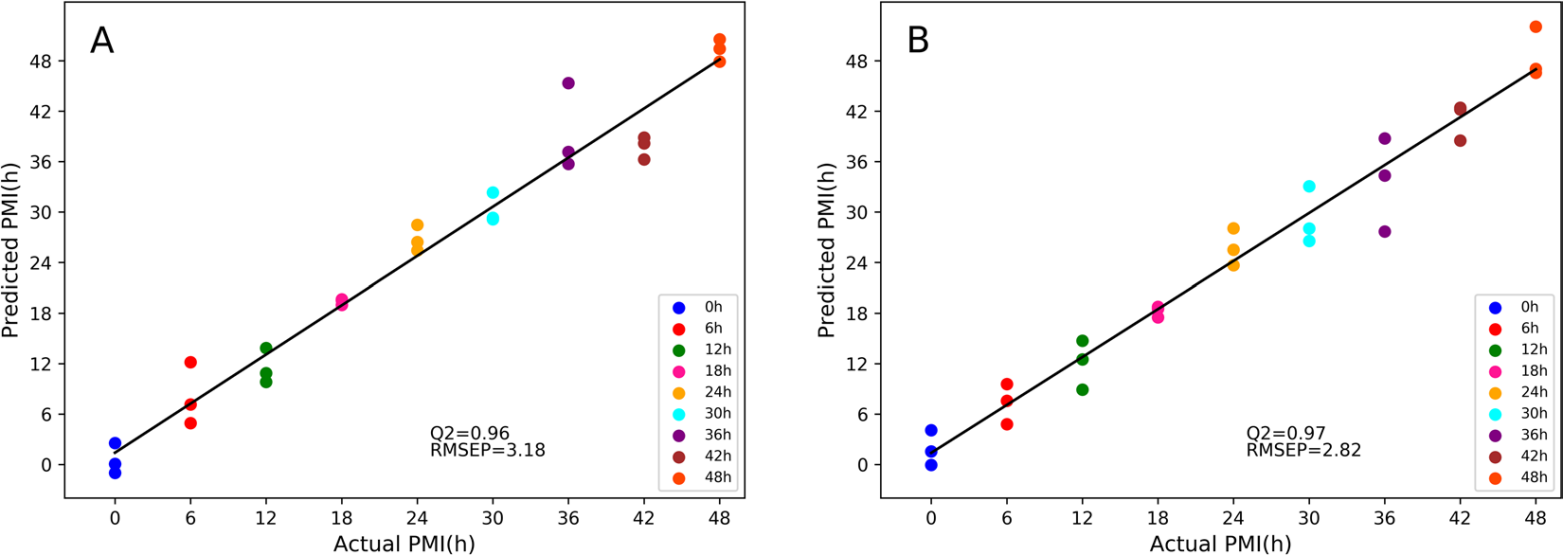
The prediction results of the PLS (**A**) and *nu*-SVM (**B**) regression models in an independent dataset which is not included in the calibration group.

**Figure 6:**
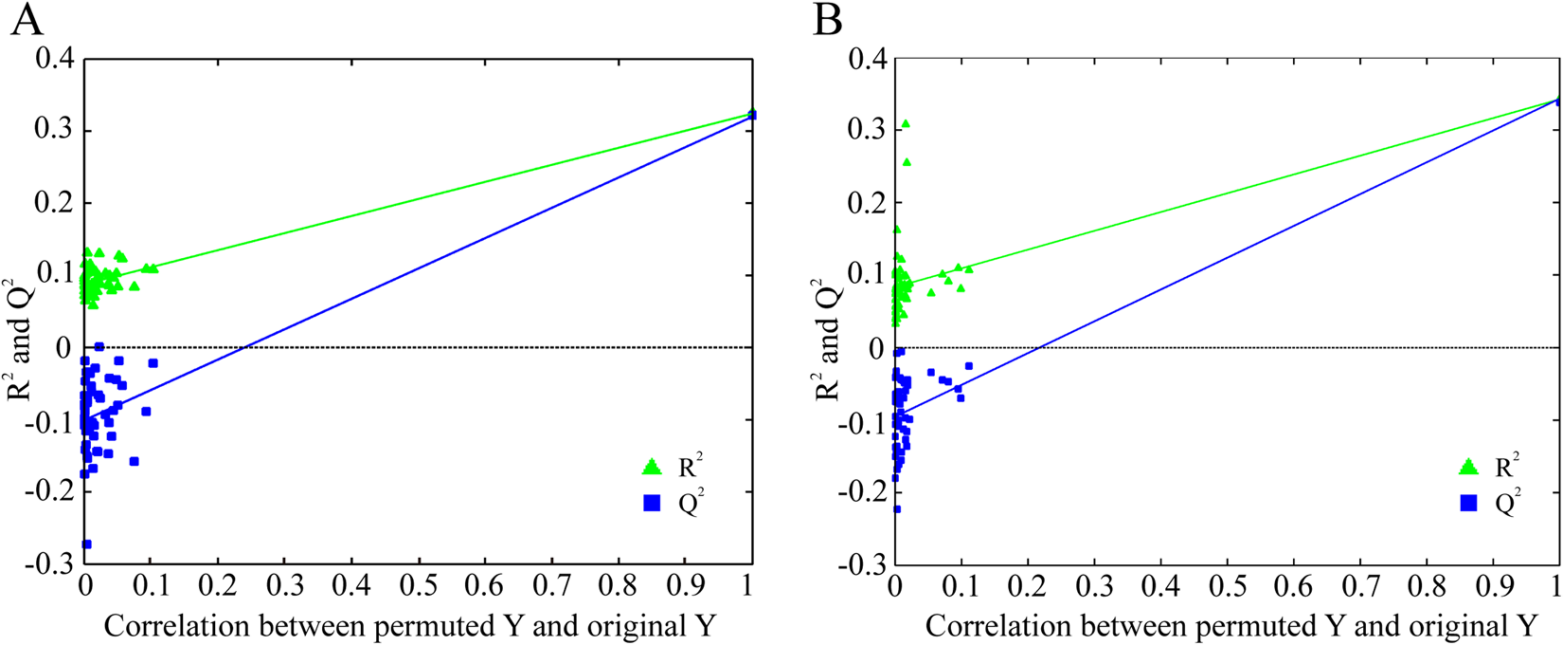
The PLS and *nu*-SVM models are validated by 50 random permutation tests as shown in (**A**) and (**B**) respectively, whose results indicate both models were acceptable.

Figure 5 shows the predicted results in the validation group using the PLS and *nu*-SVM models, respectively. The Q^2^ and RMSEP values of both models were generally close to their R^2^ and RMSCV, respectively. Moreover, a permutation test was performed to assess whether the established models were over-fitted by randomly permuting class labels and refitting new models with the same number of components as the original ones. Well fitted and meaningful models have significantly higher R^2^ and Q^2^ values than permuted data. In Fig. 6, the y-intercept values of the regression line were 0.08 and −0.1 for R^2^ for Q^2^, respectively, in the PLS model; 0.08 and −0.09, respectively, were obtained in the *nu*-SVM model. Both tests suggested that the PLS and *nu*-SVM models were reliable in predicting PMI in independent samples.

As reported previously, some metabolites and proteins in biological samples could be considered biomarkers for PMI estimation. For example, studies have shown that the concentrations of certain metabolites in plasma and muscle samples from rats are highly correlated with PMI^38,39^. Pittner and colleagues have identified degradation profiles of candidate proteins in human muscle tissues for delimiting certain periods of time postmortem, even under heterogeneous conditions such as variations in ambient temperature, age, sex, and cause of death^40^. Along with PF advantages, the current spectroscopic study suggests that the PF may be a potential medium for PMI estimation in forensic practice. Given limitations of ATR-FTIR spectroscopy, omics approaches, such as metabolomics and proteomics, are invaluable in identifying specific substances in the PF, which can greatly contribute to the discovery of new biomarkers for PMI estimation.

## Conclusion

In this work, ATR-FTIR spectroscopy was applied for the first time to acquire biochemical information in PF samples from rabbits within 48 h postmortem. Along with 2D correlation analysis, spectroscopic findings suggested that PMI-dependent changes in the PF almost solely derive from molecular vibrations of proteins, polypeptides, and amino acids, and are associated with time-ordered protein degradation. Moreover, the *nu*-SVM and PLS models were established to predict PMI, with the SVM model yielding a more satisfactory prediction according to 8-fold-cross-validation. Overall, the present findings demonstrate that ATIR-FTIR spectroscopy combined with chemometrics may be used to determine PMI, offering a promising new approach in the forensic field.

## Electronic supplementary material

Supplementary Dataset 1
